# Supplementary material for: Effects of Different Scan Projections on the Quantitative Ultrasound-Based Evaluation of Hepatic Steatosis
Source: Healthcare (Basel). 2022 Feb 14;10(2):374. doi: 10.3390/healthcare10020374 (PMC8872438; doi:10.3390/healthcare10020374)
Supplement: Supplementary file 1 [file healthcare-10-00374-s001.zip › healthcare-1544210-supplementary.pdf]

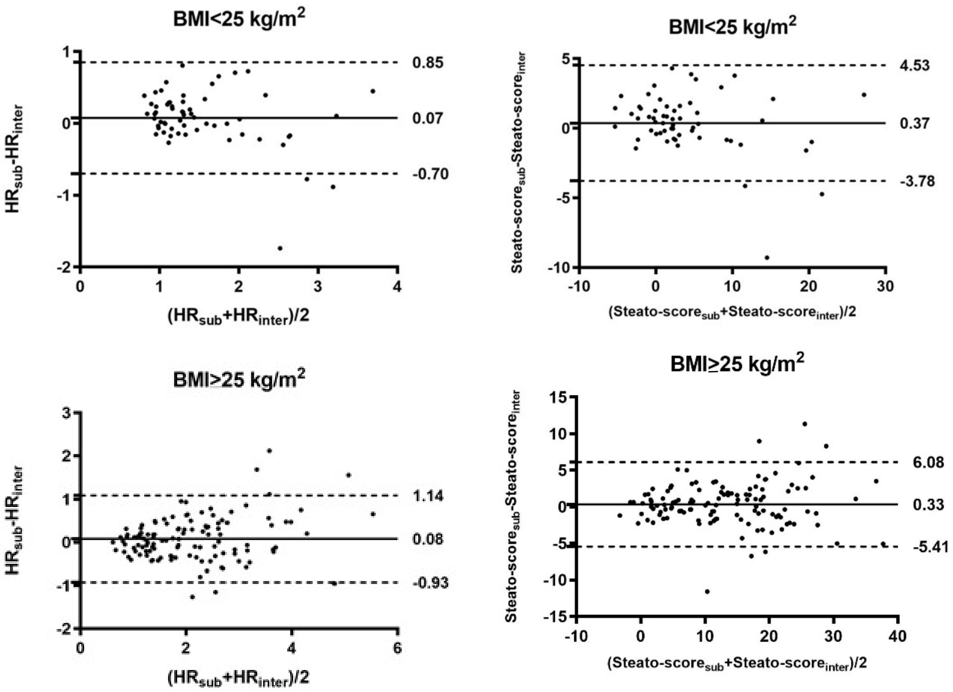

Figure S1: Bland-Altman plots of HR and Steato-score measurements assessed with two different scan views and splitting the population in two classes according BMI value (cutoff 25 kg/m2) The two dashed lines represent the limits of agreements from -1.96×SD to +1.96×SD, and the continue line represents the average difference (bias) in measurements between the two different ultrasound scans.

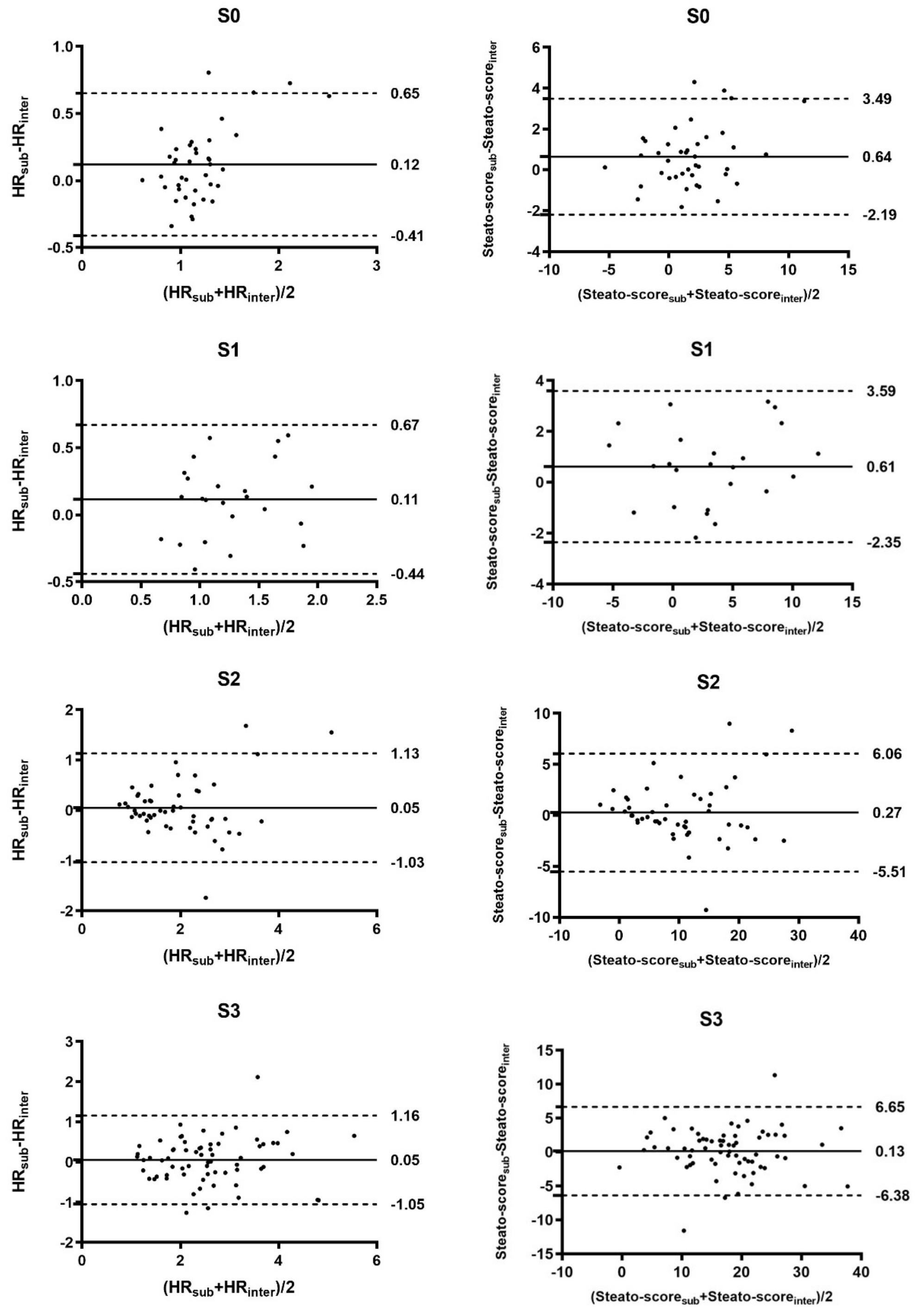

Figure S2: Bland-Altman plots of HR and Steato-score measurements assessed with two different scan views and by stratifying the whole population in four classes obtained by the qualitative classification of steatosis level (S0: absence of steatosis; S1: mild steatosis; S2: moderate steatosis; S3: severe steatosis). The two dashed lines represent the limits of agreements from -1.96×SD to +1.96×SD, and the continue line represents the average difference (bias) in measurements between the two different ultrasound scans.
